# Muscle Amino Acid and Adenine Nucleotide Metabolism during Exercise and in Liver Cirrhosis: Speculations on How to Reduce the Harmful Effects of Ammonia

**DOI:** 10.3390/metabo12100971

**Published:** 2022-10-13

**Authors:** Milan Holeček

**Affiliations:** Department of Physiology, Faculty of Medicine, Charles University, 500 03 Hradec Kralove, Czech Republic; holecek@lfhk.cuni.cz

**Keywords:** branched-chain amino acids, hyperammonemia, glutamine, glutamic acid

## Abstract

Studies from the last decades indicate that increased levels of ammonia contribute to muscle wasting in critically ill patients. The aim of the article is to examine the effects of two different causes of hyperammonemia—increased ATP degradation in muscles during strenuous exercise and impaired ammonia detoxification to urea due to liver cirrhosis. During exercise, glycolysis, citric acid cycle (CAC) activity, and ATP synthesis in muscles increase. In cirrhosis, due to insulin resistance and mitochondrial dysfunction, glycolysis, CAC activity, and ATP synthesis in muscles are impaired. Both during exercise and in liver cirrhosis, there is increased ammonia detoxification to glutamine (Glu + NH_3_ + ATP → Gln + ADP + Pi), increased drain of ketoglutarate (α-KG) from CAC for glutamate synthesis by α-KG-linked aminotransferases, glutamate, aspartate, and α-KG deficiency, increased oxidation of branched-chain amino acids (BCAA; valine, leucine, and isoleucine), and protein-energy wasting in muscles. It is concluded that ammonia can contribute to muscle wasting regardless of the cause of its increased levels and that similar strategies can be designed to increase muscle performance in athletes and reduce muscle loss in patients with hyperammonemia. The pros and cons of glutamate, α-KG, aspartate, BCAA, and branched-chain keto acid supplementation are discussed.

## 1. Introduction

Strenuous exercise and liver injury are the most common causes of hyperammonemia. It has been estimated that around 90% of patients with hyperammonemia have liver disease [1]. The causes of hyperammonemia that are not due to exercise or liver injury include inherited disorders of the urea cycle, hypovolemic shock, congestive cardiac failure, sepsis, gastrointestinal bleeding, urinary infection, inherited disorders of fatty acid oxidation, hematological malignancies, and medications used for therapy of epilepsy and cancer [2,3,4].

Several studies have reported that hyperammonemia plays a role in protein-energy wasting and increased morbidity and mortality in critically ill patients [3,4,5,6,7] and decreases concentrations of branched-chain amino acids (BCAA; valine, leucine, and isoleucine) in plasma, and glutamate, and α-ketoglutarate (α-KG) in muscles [8,9,10]. Studies performed under in vitro conditions have demonstrated that these alterations are mainly caused by the direct influence of ammonia on muscles. Exposure of isolated muscles to a medium with 0.5 mM ammonia increased leucine oxidation and glutamine synthesis, and decreased glutamate and BCAA levels in muscles [9]. In another study, exposure of myotubes to ammonia resulted in decreased myotube diameters, decreased protein synthesis, and increased expression of a range of markers of autophagy [7].

The aim of the article is to clarify similarities and differences in ammonia detoxification to glutamine and amino acid and adenine nucleotide metabolism in muscles under conditions of the different causes of hyperammonemia. In the first part, the pathways of ammonia detoxification to glutamine in a healthy man at rest are described. With this explanation as a background, the effects of two main causes of hyperammonemia, strenuous exercise and cirrhosis of the liver, will be examined. The possible strategies that might reduce the detrimental effects of ammonia on muscles will be discussed in the final part.

## 2. Muscle Ammonia and Amino Acid Metabolism at Rest

Ammonia is produced continuously during the metabolism of all organs. The main sources are amino acid metabolism, degradation of purines and pyrimidines, heme synthesis, and microbiota of the gut. Based on dietary nitrogen intake and urinary nitrogen elimination in the form of urea and ammonia can be estimated that an adult makes about 700 mmol of ammonia per day. The rise in ammonia levels in the blood, which has a toxic effect on the brain, is prevented by its temporary detoxification of glutamine. The final route of ammonia detoxification occurs via urea synthesis in the liver. Therefore, although ammonia production in the body is high, its concentration in the blood is low and does not exceed 40 µM in a healthy man at rest. Higher ammonia concentrations (0.5–1 mM) are only in the portal vein.

### 2.1. Ammonia Synthesis in Muscles

Physiologically, high amounts of ammonia are formed in skeletal muscles and its concentration in muscles is more than tenfold higher than in the blood. The main source is the deamination of AMP to ammonia and inosine monophosphate (IMP) by AMP deaminase. The reaction is activated by an increased supply of AMP produced from ADP by adenylate kinase. The greater the supply of ADP due to ATP utilization (e.g., during muscle work), the more AMP and ammonia will be formed in the sequence of three reactions:

ATP + H_2_O → ADP + Pi (ATPase)

ADP + ADP → ATP + AMP (adenylate kinase)

AMP+ H_2_O → IMP + NH_3_ (AMP deaminase)

**Figure 1:**
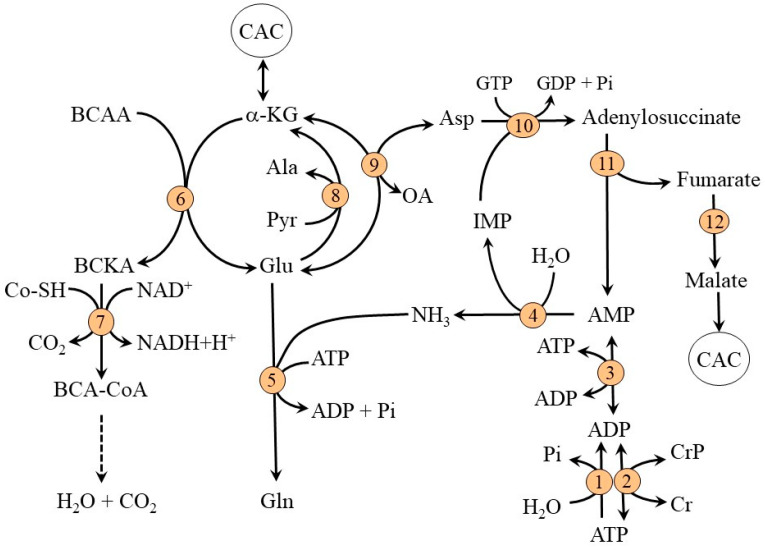
Ammonia synthesis and detoxification to glutamine in muscles. 1, ATPase; 2, creatine kinase; 3, adenylate kinase (myokinase); 4, AMP deaminase; 5, glutamine synthetase; 6, BCAA aminotransferase; 7, BCKA dehydrogenase; 8, ALT; 9, AST; 10, adenylosuccinate synthetase; 11, adenylosuccinate lyase; 12, fumarase. BCAA, branched-chain amino acids; BCA-CoA, branched-chain acyl-CoA; CAC, citric acid cycle; Cr, creatine; CrP, creatine phosphate; IMP, inosine monophosphate; OA, oxaloacetate; Pi, inorganic phosphate.

Another source of AMP is the purine-nucleotide cycle (PNC) which acts as a pathway to balance the levels of the adenine nucleotides (ATP, ADP, and AMP) via the recycling of IMP [11]. The PNC comprises reactions catalyzed by adenylosuccinate synthetase, adenylosuccinate lyase, and AMP deaminase (Figure 1). Via the PNC, the amino groups of aspartate, glutamate, and BCAA can become a source of ammonia:

BCAA + α-KG → BCKA + Glu (BCAA aminotransferase)

Glu + oxaloacetate → α-KG + Asp (AST)

Asp + IMP + GTP → adenylosuccinate + GDP + Pi (adenylosuccinate synthetase)

adenylosuccinate → fumarate + AMP (adenylosuccinate lyase)

AMP + H_2_O → IMP + NH_3_ (AMP deaminase)

Theoretically, the deamination of glutamic acid by glutamate dehydrogenase can also be a source of ammonia:

Glu + NAD(P)^+^ + H_2_O → α-KG + NH_4_^+^ + NAD(P)H

However, glutamate dehydrogenase activity in muscles is very low [12], and owing to rapid glutamate removal for ammonia detoxification to glutamine, the possibility is unlikely.

### 2.2. Ammonia Detoxification to Glutamine in Muscles

In a healthy individual, there is no significant net uptake or release of ammonia by muscles in a state of inactivity although ammonia concentration in muscles is higher than in the blood [13,14,15]. This phenomenon has two reasons.

Firstly, ammonia occurs in the body in non-ionized and ionized forms (NH_3_ ↔ NH_4_^+^). Since the pKa of ammonia is ~9.2, only 1–2% of ammonia is in the form of NH_3_ at physiological pH values. The non-ionized form diffuses easily, the ionized form is poorly soluble in fats and therefore does not diffuse through cell membrane lipids. Therefore, because the transport of ammonia between the blood and tissues is ensured mainly by NH3 diffusion, the exchange is very slow. Moreover, because the pH of the blood is higher than in muscle cells, the percentage of the non-ionized form of ammonia in the blood is higher.

Secondly, part of the ammonia produced in muscles is rapidly detoxified to glutamine in a reaction catalyzed by glutamine synthetase:

Glu + NH_3_ + ATP → Gln + ADP + Pi

Glutamine synthesized in muscles is released to the blood in exchange for the uptake of BCAA via transporter for large neutral amino acids LAT1 (SLC7A5) and metabolized predominantly in periportal hepatocytes, enterocytes, immune cells, and the kidneys to ammonia and glutamate.

**Figure 2:**
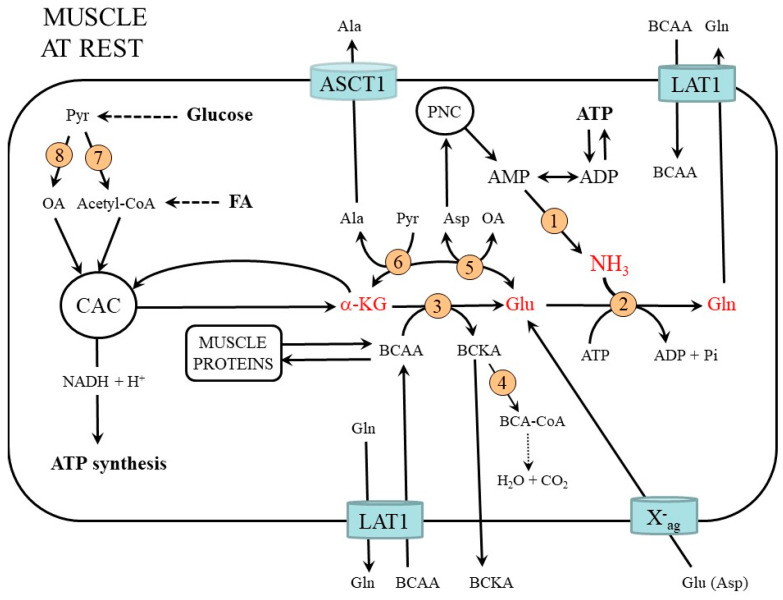
Biochemical pathways and transporters involved in ammonia and amino acid metabolism in skeletal muscle in a physiological state at rest. 1, AMP deaminase; 2, glutamine synthetase; 3, BCAA aminotransferase; 4, BCKA dehydrogenase; 5, AST; 6, ALT; 7, pyruvate dehydrogenase; 8, pyruvate carboxylase. ASCT1 (alanine, serine, cysteine, and threonine carrier 1); BCAA, branched-chain amino acids; BCA-CoA, branched-chain acyl-CoA; BCKA, branched-chain keto acids; FA, fatty acids; LAT1, large neutral amino acid transporter 1; OA, oxaloacetate; X^-^_ag_, a transporter for aspartate and glutamate.

For non-working muscles, the main source of glutamate for glutamine synthetase reaction is the blood. Most of the glutamate uptake is removed together with aspartate by a transporter specific for dicarboxylic acids termed X^-^_ag_. The system is dependent on an electrochemical gradient of sodium ions and enables a net uptake of glutamate although glutamate concentration in muscles is far higher than its concentration in plasma. Other sources of glutamate for muscles can be a breakdown of muscle proteins and synthesis by α-KG-linked aminotransferases, specifically AST (Asp + α-KG → oxaloacetate + Glu) and BCAA aminotransferase (BCAA + α-KG → BCKA + Glu). The driving force for the flux of α-KG through these reactions can be glutamate consumption by glutamine synthetase [16]. In muscles, the ALT reaction is directed toward alanine synthesis (pyruvate + Glu → Ala + α-KG) and thus can only indirectly affect glutamate production (Figure 2).

The main source of nitrogen for glutamate synthesis is the BCAA delivered to the muscles by an exchange for glutamine via LAT1 or released during the breakdown of muscle proteins. As the expression of BCAA aminotransferase (the first enzyme in BCAA catabolism) in muscles is high, whereas its expression in the liver is very low, skeletal muscle is the initial site for most of the BCAA catabolism. Glutamate synthesized by BCAA aminotransferase can then act as a source of an amino group for the synthesis of alanine from pyruvate, aspartate from oxaloacetate, or as a substrate for ammonia detoxification to glutamine (Figure 1 and Figure 2). The key enzyme in the degradation route of BCAA is BCKA dehydrogenase at the inner mitochondrial membrane. The enzyme is regulated by reversible phosphorylation mediated by a specific kinase and phosphorylase. Increased concentrations of α-ketoisocaproate (KIC, the transamination product of leucine) and decreased concentrations of ATP, NADH, and acyl-CoA derivatives activate the enzyme [16,17].

### 2.3. Compartmentation of Ammonia and Amino Acid Metabolism in Muscles

Metabolic pathways involved in ammonia synthesis and its detoxification to glutamine are compartmentalized between cytosol and mitochondria. In the cytosol are enzymes for degradation of adenine nucleotides to ammonia, glutamine synthesis, PNC, ALT, and cytosolic AST. Enzymes of the CAC, BCAA catabolizing enzymes, and mitochondrial AST are in mitochondria. The interactions between the reactions taking place in the cytosol and mitochondria are mediated by the system of inner mitochondrial membrane transporters. These are mainly aspartate-glutamate carriers (AGC) and malate-ketoglutarate carriers (MKC) that form a “malate-aspartate shuttle” and a transporter for BCAA belonging to the SLC25A family [18].

**Figure 3:**
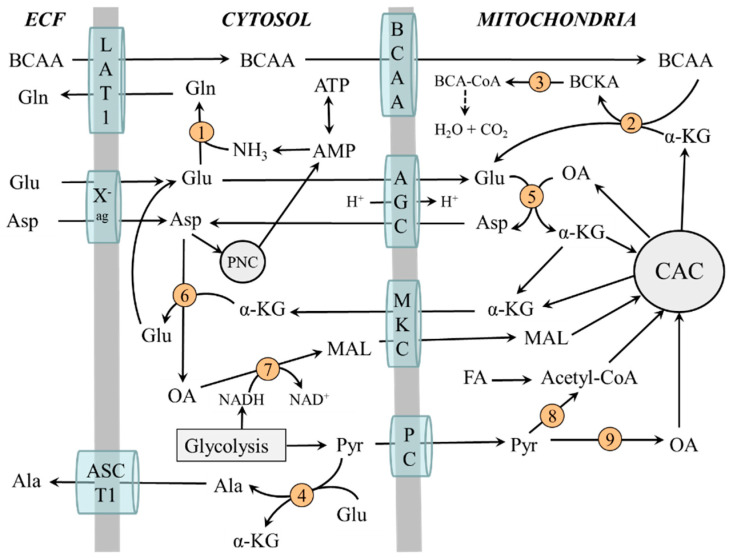
Compartmentation of ammonia and amino acid metabolism in muscles. 1, glutamine synthetase; 2, BCAA aminotransferase; 3, BCKA dehydrogenase; 4, ALT; 5, mitochondrial AST; 6, cytosolic AST; 7, malate dehydrogenase; 8, pyruvate dehydrogenase; 9, pyruvate carboxylase. AGC, aspartate-glutamate carrier; ASCT1 (alanine, serine, cysteine, and threonine carrier 1); BCAA, branched-chain amino acids; BCA-CoA, branched-chain acyl-CoA; BCKA, branched-chain keto acids; CAC, citric acid cycle; LAT1 (large neutral amino acid transporter 1); MKC, malate-ketoglutarate carrier; OA, oxaloacetate; PC, pyruvate carrier; PNC, purine nucleotide cycle; X^-^_ag_, a transporter for aspartate and glutamate.

Figure 3 shows that the BCAA are transported from cytosol to mitochondria to be used as a source of the amino group for glutamate synthesis by BCAA aminotransferase. Glutamate produced by BCAA aminotransferase or delivered to mitochondria by AGC is in mitochondria converted to α-KG and aspartate in an AST-catalyzed reaction. The α-KG can be returned to the CAC cycle or translocated to the cytosol by MKC. Aspartate is translocated by AGC to the cytosol to be used in PNC or for glutamate synthesis. Hence, the BCAAs act as the source of nitrogen for glutamate synthesis in the cytosol via aspartate synthesized in mitochondria and transported out of the mitochondria by the malate-aspartate shuttle.

### 2.4. The Role of Glycolysis and Citric Acid Cycle (CAC)

The glycolysis is the main source of pyruvate, which can be converted by pyruvate dehydrogenase to acetyl coenzyme A (acetyl-CoA) or pyruvate carboxylase to oxaloacetate. Maintaining adequate concentrations of oxaloacetate is essential for its condensation with acetyl-CoA by citrate synthase, which is recognized as a rate-limiting step in the oxidation of acetyl-CoA originating from glycolysis and fatty acid oxidation. Ammonia ions were shown to stimulate glycolysis and pyruvate carboxylase activity [19,20].

The citric acid cycle (CAC) is the main source of reducing equivalents that enter the respiratory chain, where ATP is produced, and α-KG for the synthesis of carbon skeletons of glutamate and glutamine. The flux through CAC and production of ATP and α-KG increase when glycolysis is activated. Impaired glycolysis due to diabetes or during the first days of starvation reduces the flux through the CAC and increases the BCAA levels due to decreased α-KG production and flux of BCAA through BCAA aminotransferase [21].

It should also be pointed out that the transfer of NADH formed during glycolysis into mitochondria is associated with α-KG and aspartate synthesis and their transfer from mitochondria into the cytosol by malate-aspartate shuttle:

cytosol: oxaloacetate + NADH + H^+^ → malate + NAD^+^

mitochondria: malate + NAD^+^ → oxaloacetate + NADH + H^+^

oxaloacetate + Glu → α-KG + Asp

## 3. Muscle Ammonia and Amino Acid Metabolism during Exercise

**Figure 4:**
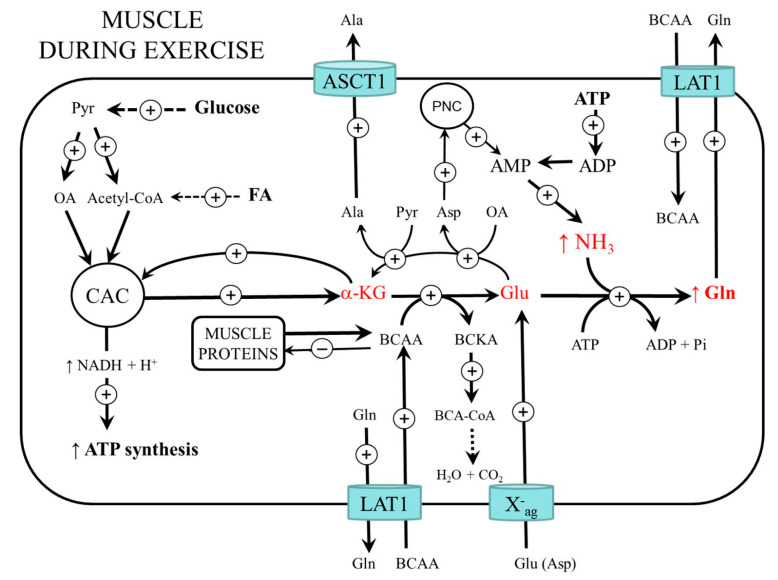
Ammonia and amino acid metabolism in muscles during exercise. Ammonia synthesis increases due to the enhanced turnover of adenine nucleotides. The main pathway of ammonia detoxification in muscles is glutamine synthesis. Increased glycolysis and CAC activity are essential for an adequate supply of α-KG and subsequent glutamate and glutamine synthesis. ASCT1 (alanine, serine, cysteine, and threonine carrier 1); BCAA, branched-chain amino acids; BCA-CoA, branched-chain acyl-CoA; BCKA, branched-chain keto acids; CAC, citric acid cycle; FA, fatty acids; LAT1 (large neutral amino acid transporter 1); OA, oxaloacetate; X^-^_ag_ (transporter for aspartate and glutamate).

Exercise greatly increases the rate of ATP utilization and deamination of AMP produced by myokinase and PNC to ammonia and IMP (Figure 4). A different situation exists during exercise at moderate intensity and exhaustive exercise. The results of several human and animal studies have demonstrated that ammonia concentrations in blood and muscles did not change or increased slightly during submaximal exercise [17,22,23,24,25]. During prolonged exhaustive exercise the rate of ATP utilization exceeds the rate of ATP synthesis, and the total adenine nucleotide pool (ATP + ADP + AMP) decreases significantly [26,27,28]. Ammonia level increases up to 250 µmol/L in plasma and up to 5 mmol/kg in muscles [14,27]. It has been shown that ammonia production is greatest during prolonged, steady-state exercise that requires 60–80% VO_2_ max [25]. It is assumed that exercise-induced hyperammonemia contributes to both central and peripheral fatigue [29].

As discussed in the previous section, the pivotal role in ammonia detoxification in muscles plays glutamate, which is the direct substrate for glutamine synthetase. Human and animal studies have shown that due to an enormous increase in glutamine synthesis, intramuscular glutamate decreases up to 80% during exercise although glutamate uptake from the blood is activated [14,27,30,31]. A role in glutamate deficiency also plays its increased utilization in alanine synthesis in the cytosol (Glu + pyruvate → α-KG + Ala) and aspartate synthesis in mitochondria (Glu + oxaloacetate → α-KG + Asp). Alanine release from muscles increases during exercise and it is well established that alanine is a physiologically important substrate for gluconeogenesis in the liver, which is increased during endurance exercise [14,30]. Aspartate formed from glutamate and oxaloacetate in mitochondria is via AGC transported to the cytosol, where it is utilized by several pathways including PNC and glutamate synthesis [11].

Both human and animal studies indicate that moderate physical exercise is associated with increased plasma glutamine concentrations [22,23,24,25,27]. However, plasma glutamine decreases during strenuous (vigorous) exercise, post-exercise recovery, and overtraining syndrome [31,32,33]. The main causes are apparently impaired glutamine synthesis in muscles and its increased use to form NH_4_^+^ by the kidneys to eliminate acidic substances formed during muscle work.

### 3.1. BCAA Metabolism

Catabolism of the BCAA in muscles increases and BCAA concentrations in plasma and muscles decrease during exercise [34,35,36]. The main causes of the increased flux of the BCAA through BCAA aminotransferase and BCKA dehydrogenase are probably decreased glutamate and ATP levels and increased KIC availability due to increased leucine transamination [16,17,22,37]. Studies in humans with ^13^C-labeled leucine showed that BCAA oxidation increased 2- to 4-fold during exercise [38,39,40].

### 3.2. Glycolysis and CAC Activity

The glycolysis and activities of pyruvate dehydrogenase and pyruvate carboxylase in muscles increase during aerobic exercise more than a hundredfold [41,42]. The result is the increased flux through the CAC and subsequent increase in NADH and ATP production. In human muscle, the flux through the CAC increased ~70-fold during submaximal exercise and was ~100-fold higher than at rest at exhaustion [43]. It was shown that net hindlimb glutamine efflux increased in response to glucose administration in exercised but not sedentary dogs [44].

It can be assumed that the decrease in glucose oxidation and CAC activity due to oxygen deprivation during strenuous exercise will decrease α-KG supply for glutamate synthesis and subsequent ammonia detoxification to glutamine. Some studies, but not all, have shown that whereas the concentrations of most intermediates of the CAC increased in human muscles during exercise, the concentration of α-KG decreased [43,44,45,46].

### 3.3. Protein Metabolism during and after Muscle Work

During exercise, muscle protein catabolism is activated as evidenced by the decreased content of muscle proteins in rodents after prolonged physical activity [47,48]. The main cause is probably depressed protein synthesis, the reports of the effect of exercise on protein degradation are not consistent [38,49,50]. It is very likely that the role in decreased protein synthesis plays ATP and BCAA deficiency.

It should be noted that although intense muscle work induces loss of muscle proteins, regular physical activity does not lead to a decrease in muscle mass, but vice versa. It has been shown that during the post-work regeneration period, protein synthesis is activated in the muscles in parallel with the formation of energy stores, and the proliferation and differentiation of satellite cells [51,52].

## 4. Ammonia Metabolism in Liver Cirrhosis

This article deals only with decompensated cirrhosis with manifestations of hepatic encephalopathy and elevated blood ammonia levels. The most common causes are viral hepatitis, alcoholism, biliary obstruction (biliary cirrhosis), and chronic heart failure (cardiac cirrhosis). Regardless of the etiopathogenesis, the cause of hyperammonemia is portal-systemic shunts and/or impaired ammonia detoxification in the liver to urea.

In cirrhosis, similar to strenuous exercise, ammonia detoxification to glutamine in muscles is activated. A marked increase in glutamine and a decrease in glutamate, α-KG, aspartate, alanine, and BCAA in muscles has been found in a rat model of liver cirrhosis [10]. In plasma, glutamine usually rises, but there are also data on its unchanged or decreased concentration [10,53,54,55,56].

**Figure 5:**
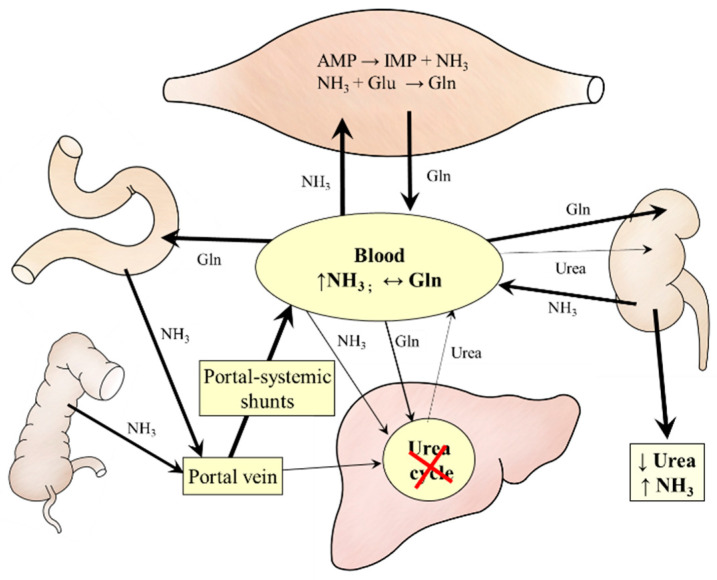
Ammonia and amino acid metabolism in cirrhosis. Ammonia levels increase due to impaired urea synthesis in the liver, portal-systemic shunts, and increased glutamine catabolism in visceral organs.

Unlike exercise, the temporary ammonia detoxification to glutamine in the muscles is not very effective. Several studies reported mitochondrial impairment in the muscles of patients with liver cirrhosis and demonstrated that cirrhosis depletes CAC intermediates, especially α-KG, and ATP in muscles [10,57,58,59]. It can be supposed that insulin resistance, depletion of glycogen stores, decreased glycolysis, and increased drain of α-KG from the CAC (cataplerosis) play a role in impaired flux through the CAC and mitochondrial dysfunction. Moreover, unlike exercise, much of the glutamine released to the circulation is not detoxified to urea but catabolized to form ammonia (Figure 5). A vicious cycle, in which ammonia detoxification to glutamine in muscles leads to increased glutamine degradation to ammonia in visceral tissues that further emphasizes the increase in blood ammonia levels, has been postulated [60].

### 4.1. BCAA Metabolism

**Figure 6:**
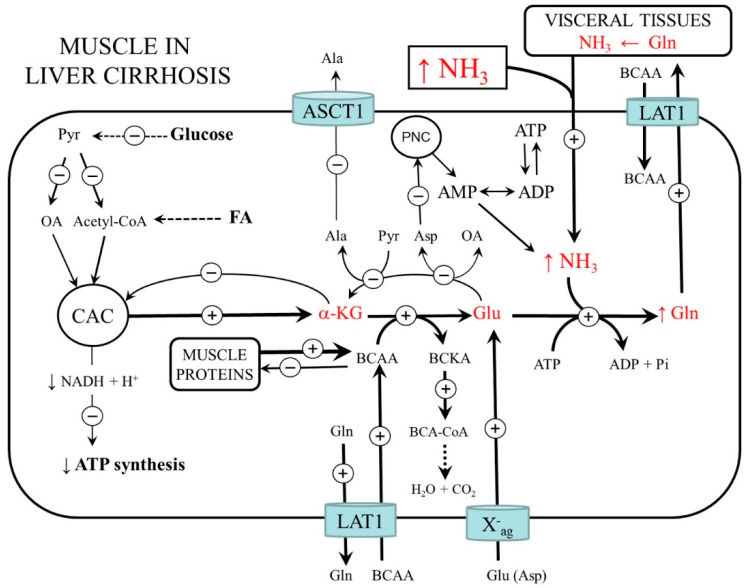
Ammonia and amino acid metabolism in cirrhosis. Increased ammonia detoxification to glutamine in muscles results in BCAA deficiency, cataplerosis (drain of α-KG from CAC), and mitochondrial dysfunction. Due to the limited activation of glycolysis and mitochondrial dysfunction, the detoxification of ammonia is less efficient than during exercise. ASCT1 (alanine, serine, cysteine, and threonine carrier 1); BCAA, branched-chain amino acids; BCA-CoA, branched-chain acyl-CoA; BCKA, branched-chain keto acids; CAC, citric acid cycle; FA, fatty acids; LAT1 (large neutral amino acid transporter 1); OA, oxaloacetate; X^-^_ag_ (transporter for aspartate and glutamate).

Enhanced rates of BCAA oxidation in liver cirrhosis have been demonstrated both in animals and human subjects [61,62]. Similar to heavy exercise, the main causes are increased flux through BCAA aminotransferase due to glutamate deficiency and increased BCKA dehydrogenase activity due to ATP deficiency. The increased requirements of the BCAA in muscles are fulfilled by their enhanced uptake from the extracellular fluid by exchange with glutamine via L-system (SLC7A5; LAT1). Therefore, the final cause of decreased BCAA levels in the blood in liver cirrhosis is their increased influx to muscles (Figure 6).

### 4.2. Protein Metabolism

Liver cirrhosis is a typical protein-wasting disorder. The loss of muscle mass limits the body’s ability to detoxify ammonia to glutamine, reduces muscle strength and their exercise capacity, and increases the risk of several complications. The prevalence of cachexia in patients with liver cirrhosis is about 50% [63].

The pathogenesis of muscle wasting is complex. Unless cirrhosis is complicated by a systemic inflammatory response, the dominant cause is a decrease in protein synthesis due to anorexia, maldigestion, and malabsorption of nutrients, and some neurohumoral changes (e.g., decreased IGF-1 production). In the phase of disease progression and under the influence of inflammatory factors, especially cytokines, increased protein turnover and activation of proteolysis can be observed. It is likely that hyperammonemia-induced changes, especially mitochondrial dysfunction and BCAA deficiency, play a role in protein-energy wasting regardless of whether the cirrhosis is accompanied by an inflammatory reaction or not.

## 5. Possibilities to Reduce the Harmful Effects of Ammonia on Muscles

All aminotransferase reactions involved in ammonia detoxification to glutamine in muscles are reversible, their Km is above usual tissue concentrations of their reactants, and are sensitive to the supply of α-KG, glutamate, aspartate, BCAA, and BCKA [16]. Removal of glutamine can also affect the flux through aminotransferase reactions, and subsequently the flux through the CAC, mitochondrial function, and BCAA oxidation [64]. Therefore, several strategies that might attenuate the detrimental effects of hyperammonemia on amino acid metabolism, mitochondrial function, and protein metabolism in muscles can be hypothesized.

### 5.1. Glutamate

Glutamate is effectively taken up by resting and active muscle and an increase in plasma glutamate levels can replenish its deficiency observed in muscles after strenuous exercise and in liver cirrhosis. Several studies have shown that ingestion of monosodium glutamate (~100–150 mg/kg) increases plasma glutamate and glutamine levels up to 700–800% [65,66,67]. Graham et al. [66] demonstrated that monosodium glutamate ingestion by resting humans can elevate intramuscular glutamate concentration by ~40%.

Glutamate administration increased exercise duration in a dose-related way when given intravenously to patients with stable angina pectoris [68]. In the 1950s, a number of studies were carried out on the treatment of hepatic encephalopathy with L-glutamic acid and several publications have appeared with somewhat conflicting conclusions [69,70,71]. It was finally proved that L-glutamic acid has only a transient effect and that a temporary fall in ammonia levels occurring during its administration is followed by a rise to pretreatment levels as soon as glutamate administration was discontinued [72].

It should be noted that the absorption of dietary glutamate is limited by its extensive metabolisms in enterocytes [73,74]. Therefore, decreased ammonia and elevated glutamate, alanine, and glutamine levels in plasma and muscles in subjects administered orally by monosodium glutamate are partly due to alanine synthesis from ingested glutamate in enterocytes and decreased intestinal uptake of glutamate and glutamine from the blood.

### 5.2. α-KG

The replenishment of α-KG in muscles may activate glutamate synthesis and ammonia detoxification to glutamine and attenuate the drain of α-KG from the CAC. Moreover, orally administered α-KG is oxidized by enterocytes and in this way may suppress glutamine catabolism and ammonia production by the gut [75].

The shortcoming of α-KG as a supplement is that it penetrates little across the plasma membrane. Therefore, more effective might be its cell-permeable derivatives, e.g., dimethyl-α-KG, which have been shown to reverse the low levels of CAC intermediates and ATP content in myotubes due to hyperammonemia [59].

The adverse effect of enhanced α-KG supply can be increased BCAA catabolism and worsening of BCAA deficiency in the body. Therefore, BCAA supplementation should be recommended when the α-KG level in muscles is replenished artificially.

### 5.3. BCAA

A rational basis for use of the BCAA is their decreased concentration in plasma both during exercise and in liver cirrhosis and their pharmacological properties, the particularly positive effect of leucine and several BCAA metabolites, such as BCKA and beta-hydroxy-beta-methylbutyrate, on protein metabolism [76,77,78]. It is supposed that correction of the decrease in the ratio of the BCAA to aromatic amino acids (tryptophane, phenylalanine, and tyrosine) in plasma reduces the synthesis of serotonin and false neurotransmitters in the brain and subsequently delays the onset of fatigue during muscle work and improves brain function in patients with hepatic encephalopathy [79]. Moreover, it was shown that BCAA supplementation increases ammonia detoxification to glutamine in muscles and prevents the decrease in the plasma glutamine level during long-term exercise [80,81].

Unfortunately, no valid scientific evidence supports the commercial claims that BCAA has a beneficial effect on muscle performance [23,82,83,84,85]. Nor the results of the clinical trials examining the therapeutic effects of the BCAA on encephalopathy in cirrhosis are consistent [86,87]. The cause is probably some adverse effects of the BCAA administration. These include increased ammonia levels due to increased flux through PNC in muscles and increased glutamine catabolism in visceral tissues, and the drain of α-KG from the CAC (cataplerosis) due to increased glutamate synthesis [83,84,85,88,89].

### 5.4. Branched-Chain Keto Acids (BCKA)

**Figure 7:**
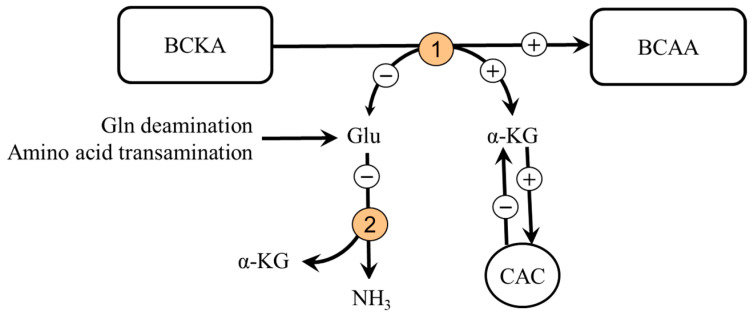
Supposed effects of BCKA supplementation on BCAA and ammonia synthesis. 1, BCAA aminotransferase; 2, glutamate dehydrogenase.

Significant amounts of data demonstrate protein anabolic effects of the BCKA [76,90,91,92,93], that BCKA can be aminated to original amino acids in several tissues, particularly in the kidneys, gut, and the liver [90,94], and that their administration decreases ammonia production [95,96,97]. Amination of the BCKA in subjects with impaired liver function was demonstrated by the increased utilization of labeled KIC (ketoleucine) for the synthesis of proteins in a rat mode of liver injury and portal-systemic shunting [98]. It can be supposed that the BCKA amination is facilitated by enhanced glutamine availability and decreased BCAA levels, as occurs in liver cirrhosis and intensive exercise. In addition, the BCKA administration should via increased synthesis of α-KG attenuate its drain from the CAC (cataplerosis) and decrease ammonia production in visceral tissues due to the shift of glutamate from glutamate dehydrogenase reaction towards BCAA aminotransferase (Figure 7).

The studies examining the effects of ketoanaloguesl of amino acids in the form of Ketosteril^®^ (Fresenius Kabi, Bad Homburg, Germany) have demonstrated that their acute use can decrease hyperammonemia induced by exercise [99,100,101,102], others reported beneficial effects in therapy of portal-systemic encephalopathy [93,94,95,96,97,98,99,100,101,102,103,104,105,106,107]. Unfortunately, the limitations of all these studies are the use of mixtures for subjects with renal insufficiency, which may not be suitable for patients with liver cirrhosis. Studies examining specifically the effects of the BCKA are not available.

### 5.5. Aspartic Acid

Aspartate supply can increase the formation of glutamate in muscles during aspartate transamination by cytosolic AST (Asp + α-KG → oxaloacetate + Glu) and subsequent detoxification to glutamine. Beneficial effects of aspartate supplementation can be mediated also by its role in the malate-aspartate shuttle (delivery of NADH produced during glycolysis into mitochondria) and the stimulating effect on urea formation in the liver. Several studies reported that aspartate levels in plasma and muscles decrease after exhausting exercise and in liver cirrhosis [8,10,34,108,109].

The results of the studies examining the ergogenic effects of aspartate are not convincing [110]. More promising seems to combine aspartate with minerals, amino acids (e.g., ornithine, arginine, asparagine), and other substances, such as carnitine [111,112]. Several studies reported ammonia-lowering effects of LOLA (L-ornithine L-aspartate), an agent employed for the treatment of hepatic encephalopathy [113].

### 5.6. Glutamine

During strenuous exercise, the utilization of glutamine often exceeds its synthesis, and glutamine concentration decreases in both plasma and tissues [31,32,33]. It has been shown that glutamine deficiency activates BCAA oxidation and inhibits protein synthesis in muscles and contributes to impaired immune function and gut integrity in severe illness [64,81,114,115]. Studies in athletes reported that glutamine supplementation can attenuate immunodepression, favor muscle strength recovery, and decrease the incidence of sickness after exhaustive exercise [116,117,118]. It needs to be emphasized that glutamine administration should be avoided in patients with liver cirrhosis because most glutamine is metabolized to form ammonia.

### 5.7. Ammonia Removal

Hemodialysis and several metabolic scavengers including sodium phenylbutyrate, glycerol phenylbutyrate, ornithine phenylacetate, AST-120 (spherical carbon adsorbent), and sodium benzoate have been proposed to reduce ammonia levels [119,120,121,122]. Unfortunately, most studies evaluating the effects of these therapies examined only the effects on symptoms of encephalopathy. The possible effects on muscles have not been investigated although some ammonia scavengers may exert adverse side effects. An example is a phenylbutyrate, which increases glutamine excretion from the body by urine. It was demonstrated that phenylbutyrate increases BCAA catabolism and proteolysis in muscles and impairs the regeneration of the liver [123,124].

## 6. Summary

### 6.1. Similarities and Differences in Ammonia Metabolism in Muscles during Exercise and in Liver Cirrhosis

Results of the studies presented in this article demonstrate that some changes in muscles caused by elevated ammonia levels due to increased ammonia production during strenuous exercise and due to insufficient ammonia detoxification to urea in liver cirrhosis are similar. In both, there is protein-energy wasting, increased ammonia detoxification to glutamine, the drain of α-KG from CAC for glutamate synthesis by α-KG-linked aminotransferases, glutamate and α-KG deficiency, and increased BCAA oxidation in muscles.

Apart from the fact that the changes during exercise are temporary and the changes in liver cirrhosis are long-term or permanent, the main differences are in glycolysis and mitochondrial function. During muscle work, the glycolysis, the CAC activity, and ATP turnover in muscles increase whereas in liver cirrhosis there is a decrease due to impaired function of mitochondria and insulin resistance. Another important difference is in the fate of glutamine released from the muscles into the blood. During muscle work, glutamine acts as a non-toxic form of ammonia transport to the liver for urea synthesis and it is utilized by several tissues, such as the kidneys, immune cells, and enterocytes. In liver cirrhosis, a vicious cycle in which glutamine synthesized from ammonia in the muscles is catabolized to ammonia in visceral tissues, resulting in a further increase in ammonia levels, is activated.

### 6.2. Considerations on How to Reduce the Harmful Effects of Ammonia on Muscles

The disturbances in amino acid metabolism induced by enhanced ammonia detoxification to glutamine in muscles indicate some possibilities for improving muscle performance and the prevention and muscle wasting due to hyperammonemia of both hepatic and non-hepatic origin. It is obvious that the rational basis should be the correction of glutamate, α-KG, BCAA, and aspartate deficits:

- Glutamate and α-KG—administration could promote ammonia detoxification to glutamine, reduce α-KG drain from CAC, and increase the supply of reduced nucleotides for respiratory chains in mitochondria. However, studies examining the effects of glutamate on muscles in humans with hyperammonemia are rare [68]. Glutamate administration may be detrimental in liver cirrhosis due to the increased synthesis of glutamine that is catabolized to ammonia in visceral tissues.
- BCAA—administration could increase ammonia detoxification to glutamine in muscles and correct BCAA deficiency in the blood. Potential adverse effects include increased ammonia production via the PNC and drain of α-KG from the CAC [83,84,85,88,89,125,126].
- BCKA—administration could correct BCAA deficiency in the blood, improve muscle protein balance, decrease the drain of α-KG from the CAC (cataplerosis), and decrease ammonia production in glutamate dehydrogenase reaction. The BCKA administration is not associated with an increase in ammonia levels observed after BCAA administration [95,96,97]. Unfortunately, studies examining specifically effects of BCKA supplementation are not existing.
- Aspartate—can increase the formation of glutamate in muscles via cytosolic AST. Its combination with other substances appears to be promising [111,112].
- Glutamine—can exert benefits after strenuous exercise and in over-training syndrome [116,117,118]. However, glutamine should be avoided in patients with liver cirrhosis, in which detoxification of ammonia produced from glutamine by glutaminase and glutamate dehydrogenase reactions is impaired.
- Ammonia removal—reports of increased BCAA oxidation, negative protein balance in muscles, and impaired liver regeneration following phenylbutyrate administration [123,124] indicate the need to investigate the side effects of all therapies used to reduce ammonia levels.

## 7. Conclusions

We are not aware of any work that compares the effects of exercise and cirrhosis on ammonia and amino acid metabolism in muscles. It is concluded that:

- The similarities in the influence of increased levels of ammonia due to strenuous exercise and liver cirrhosis on BCAA, glutamate, α-KG, aspartate, and adenine nucleotide metabolism in muscles indicate that ammonia can significantly contribute to muscle wasting regardless of the cause of its increased levels.
- Similar strategies can be designed to reduce the adverse effects of ammonia on the muscle, increase muscle performance in athletes, and reduce muscle loss in patients with hyperammonemia.
- To avoid harmful effects of ammonia on muscles, ammonia and plasma amino acid concentrations should be monitored in individuals with diseases in which ammonia levels are often elevated.
- Systematic investigation is needed to understand better the relationships between ammonia metabolism and the metabolism of other amino acids in the pathogenesis of muscle wasting due to increased ammonia levels.
